# Can training of health care workers improve data management practice in health management information systems: a case study of primary health care facilities in Kaduna State, Nigeria

**DOI:** 10.11604/pamj.2018.30.289.15802

**Published:** 2018-08-24

**Authors:** Bilkisu Nwankwo, Mohammed Nasir Sambo

**Affiliations:** 1 Department of Community Medicine, College of Medicine, Kaduna State University, Kaduna, Nigeria; 2 Department of Community Medicine, College of Medicine, Ahmadu Bello University, Zaria, Nigeria

**Keywords:** Data management, practice, healthcare workers, training

## Abstract

**Introduction:**

Reliable and accurate public health information is essential for monitoring, evaluating and improving the delivery of healthcare services. The objective of this study was to assess the effect of training health care workers on data management practice in health management information systems in primary health care (PHC) centers in Kaduna state.

**Methods:**

The study was quasi-experimental with baseline, intervention and end point components. It was carried out in two local government areas, a study and a control. Eleven PHC facilities were selected in each LGA. The intervention was carried out among 76 PHC workers in the study LGA. Data were collected using a health facility checklist and a focused group discussion (FGD) guide. Data analysis was done using SPSS version 20.0 and statistical significance of the difference between baseline and end-line data were determined using chi-square or fisher's exact test where applicable at p < 0.05.

**Results:**

There was a statistically significant increase in completeness of reporting (p = 0.02), overall accuracy rate (p < 0.001), timeliness rate of reporting (p = <0.001) and feedback (p = 0.012). No improvement was found in the control group. During the baseline FGDs, PHC workers in both study and control LGAs expressed difficulty in filling registers/forms, data analysis and use of data. At end point, those in the study LGA said their practice had improved but those in the control LGA still expressed difficulty in data management.

**Conclusion:**

Health management information system training achieved an improvement in the data management practice of PHC workers. In-service training and re-training should be done to improve data management practice of health workers.

## Introduction

Health information is the foundation of public health. Accurate public health information is essential for monitoring and evaluating health and also for improving the delivery of healthcare services and programs [1,2]. Health management information system (HMIS) is designed to integrate data collection, processing, reporting and use for the improvement of patient health services, effectiveness and efficiency through better management of patient data at all levels of implementation [3]. The global shift from curative to preventive care, from centralized to decentralized healthcare, from specific project approach to a comprehensive sectoral approach, has necessitated the restructuring of fragmented health information system into single comprehensive health and management information system [4]. Data management is a set of procedures for the collection, storage, processing and compilation of data [5]. This is creating information by an organization that is timely, accurate, clear, concise and presented in a way that is appropriate for the users' needs. Relevant, accurate, quantitative and qualitative data that are collected and used in a timely and efficient manner are essential for delivery of patient/consumer care and management of services [6]. The usefulness of many HMIS are determined in part by their timeliness. Epidemiologic surveillance data that are months old are obviously of limited value in helping the health system to recognize and respond to the threat of infectious disease outbreaks. A HMIS that cannot collect, analyze and report on data within a time frame of the data's usefulness (within the time frame of the decision making processes) is of little value or effect [7, 8].

The HMIS is not to be seen only as a mechanism for collecting information and passing it to successively higher levels. Information should be used at the level at which it is collected [9]. Data quality and data use are interrelated. Poor quality data will not be used and because they are not used, the data will remain of poor quality; conversely, greater use of data will help to improve their quality, which will in turn lead to more data use [10]. Local use of information can improve data quality as correction can easily be made at the point of collection in addition to helping health providers monitor their performance [7]. Feedback of information to the providers of health data is essential. The success of any management information system depends on the feedback of data collected. It is the backbone of a sound information system and provides incentives ideally for providing accurate and up to date data [11]. In order to collect valuable, relevant and cost effective data, primary health care (PHC) workers should learn to appreciate the importance of statistical data and develop the skills needed to collect necessary and useful data [12]. Training them on data management will motivate and empower them to recognize the importance of gathering accurate and reliable informations. Studies in Nigeria have shown the need for training of primary health care workers on HMIS to improve data quality [11-14]. There is a paucity of interventional studies in this regard especially in Northern Nigeria. Therefore, this study is aimed at assessing the effect of training on data management practice on HMIS among primary health care workers in Kaduna State, Nigeria.

## Methods

The study was conducted in Giwa and Kaduna North local government areas (LGAs), which are semi urban communities in Kaduna state, North West Nigeria. This study was quasi experimental with baseline, intervention and end point components. The sample size for PHC workers for training was determined using the formula for comparison of proportions in the baseline and end point components of a study [15], with the probability score at 95% interval and a precision level of 5%.

$$\text{n}=\left({\text{Z}}_{\text{1-α}}{+\text{Z}}_{\text{1-β}}\right)\left\{\left({\text{p}}_{1}{\text{q}}_{1}{+\text{p}}_{2}{\text{q}}_{2}\right)/{\left({\text{p}}_{1}{-\text{p}}_{2}\right)}^{2}\right\}$$

**Where n**= minimum sample size

for each group, Z_1-α_= standard normal deviate corresponding to the 95% confidence interval,

for the study i.e. 1.96, Z_1-β_= standard normal deviate corresponding to 80% power of study,

i.e. 0.840, p_1_ = proportion of health care workers' practice of data recording and reporting at baseline from a previous study,

i.e. 0.519 [16], p_2_ = expected level of practice at the end of the study.

An increase of 10% (i.e. 0.10) in data management practice was expected at the end of the study. A minimum sample size of 69 PHC workers was thus calculated for each group. This increased to 76 PHC workers when provision of 10% non-response rate was made. PHC workers who were involved in data management were included in the study. Health workers on leave and those due for retirement during the study period were excluded from the study. Multistage sampling was used to select PHC facilities and participants. Two LGAs (Giwa and Kaduna north) were selected from a list of the 23 LGAs in Kaduna State and by the toss of a coin, Giwa became the study LGA and Kaduna North the control LGA. From the list of PHC facilities, one PHC facility was selected from each of the 11 wards in the study and control LGAs using simple random sampling by balloting. In total, 22 PHC facilities were selected. Giwa LGA has 17 PHCs while Kaduna North has 16 PHCs. Each ward had a minimum of one PHC and a maximum of 3. Those that had one had that one PHC used automatically for the study and the ones that had more than one, had one chosen from them using simple random sampling by balloting. At each of the selected PHC facilities, eligible health care workers were selected using proportionate sampling method to obtain the sample size. Those included in the study were health workers involved in vaccination in the selected PHCs. Four FGDs were carried out in each LGA (study and control). Two were done at baseline and at end point, one with nurses and the other with community health extension workers (CHEWS). Eight nurses and eight CHEWS were selected from each LGA. They were purposively selected based on cadre to ensure some degree of homogeneity in each group.

The quantitative data were collected with the aid of a health facility checklist. The variables measured in the health facility checklist were the availability of forms and registers for data recording and reporting, completeness and correctness of data, accuracy rate of the information recorded, analysis of data, presentation of data, local use of data, timeliness of reporting and feedback. The daily out-patient, antenatal clinic attendance, daily immunization registers and the health facility monthly summary forms were reviewed. The qualitative data were collected with the aid of a FGD guide. The FGD guide asked questions on filling of registers/ forms, local use of data, feedback, problems they have with data management and possible solutions. Data collection was done at baseline using both quantitative and qualitative methods. Four training modules which were adapted from *"a manual for strengthening HMIS data quality"* [17], *"use of information training manual"* [18], *"health management information system information: facilitator's guide for training of trainers"* [19], and *"HMIS information use training manual"* [20] were used for the training intervention. The first module covered an overview of HMIS while data quality, health facility HMIS recording and reporting system, analysis, presentation, information use and feedback were the second, third and fourth modules, respectively. Permission was obtained from the LGA chairman through the PHC coordinators of both local government areas and all the supervising heads of the selected PHC facilities before the study was conducted. Information about the study was provided to each participant and their anonymity and the confidentiality of their responses, voluntary participation and right to withdraw at any stage was emphasized, following which informed written consent was obtained from each participant. The PHC workers were divided into two groups of 38 participants each. This was to ensure ease of interaction. The training was for a total of eight days per group, carried out for two hours per day (2pm to 4pm) on two days per week for four consecutive weeks. A module was taken per week. Each group had 8 training sessions and 38 participants were trained per session. The training was delivered by the researcher, with support from three trained research assistants. The methods employed in the training included interactive sessions using flip charts and group exercises. The venue of the training was the multipurpose hall in Giwa LGA which was easily accessible to all the selected PHC facilities.

Data were collected 3 months after the training intervention for 5 days from both study and control groups using the same data collection tools and research team used to collect the baseline data. The data were analyzed using statistical package for social sciences (SPSS) software version 20.0 and statistical significance of the difference between baseline and end-line data were determined using chi-square or fisher's exact test where appropriate. The level of significance was set at p < 0.05. Data completeness was when all the relevant data elements in a patient/client register were filled [21]. Data correctness was when there were no errors (e.g duplicate data, capture in the wrong box) detected in the register [21]. Accuracy rate was determined by comparing the data recorded in the summary forms (HMIS 001) with those in the facility registers. An individual data element was considered accurate when the value recorded in the summary forms lays within 10% of the corresponding value calculated by the study team from the registers. Larger differences were considered as errors rather than inaccuracies [21]. Timeliness of reporting is when health facility summary forms are remitted to the health department of the LGA not later than 2 weeks following the month of reporting [14]. Completeness of reporting is the number of reports received divided by the number of reports expected for a specified time period (3 months in this study) [22, 23].

$$\text{Formula}\;\text{for}\;\text{completeness}\;\text{of}\;\text{reporting}\;\text{(\%)}\;=\frac{\text{Total}\;\text{number}\;\text{of}\;\text{reports}\;\text{received}}{\text{Total}\;\text{number}\;\text{of}\;\text{reports}\;\text{expected}}\text{*100}$$

**Timeliness rate of reporting:** This was measured by the number of reports sent on time to the LGA health department divided by the number of reports expected to be sent [24].

$$\text{Formula}\;\text{for}\;\text{timeliness}\;\text{rate}\;\text{(\%)}\;=\frac{\text{Number}\;\text{of}\;\text{reports}\;\text{sent}\;\text{to}\;\text{LGA}}{\text{Total}\;\text{number}\;\text{of}\;\text{reports}\;\text{expected}}\text{*1}00$$

**Data use**: This was assessed from the reports of staff meetings in the health facilities and the reports of the matrons-in-charge [**18**].

## Results

Eleven PHC facilities were selected from the study and control LGAs making a total of 22. The mean age of healthcare workers in the study health facilities was 34.6 ± 8.3 years and 33.7 ± 8.4 years for the control health facilities. Junior community health extension workers (JCHEWs) constituted a greater proportion of respondents in both the study (34.3%) and control (27.1%) health facilities. At baseline, majority of the healthcare workers in both the study and control LGAs had not been trained on HMIS (92.1% and 90.8% respectively). Health facility summary forms, ANC, OPD and daily immunization registers were all available at baseline and end point in both study and control health facilities. The proportion of health facilities with completely filled OPD registers in the study LGA increased from baseline levels of 1 (9.1%) to 4 (36.4%) at end point. No health facility in the control LGA had completely filled OPD registers both at baseline and at end point. At baseline no health facility in the study LGA had completely filled ANC registers. At the end of the study it increased to 3 (27.3%), while the proportion of health facilities with completely filled ANC registers at end point remained the same with that of the baseline in the control LGA, 1 (9.1%). At baseline no health facility in the study LGA had completely filled daily immunization registers. At end point it increased to 3 (27.3%). No health facility in the control LGA had completely filled daily immunization registers both at baseline and at end point. The proportion of health facilities in the study LGA with completely filled HMIS 001 forms increased from 1 (9.1%) to 4 (36.4%). The percentage of health facilities with completely filled HMIS form 001 was 1 (9.1%) in the control LGA at both baseline and end point (Table 1). The proportion of health facilities in the study LGA with correctly filled ANC registers increased from 2 (18.2%) at baseline to 5 (45.5%) at end point. No health facility in the control LGA had correctly filled ANC registers both at baseline and at end point (Table 1). At baseline no health facility in the study LGA had correctly filled daily immunization registers. At end point it increased to 3 (27.3%). No health facility in the control LGA had correctly filled daily immunization registers both at baseline and end point. At baseline no health facility in the study LGA had correctly filled OPD registers. At end point it increased to 2 (18.2%). No health facility in the control LGA had correctly filled OPD registers both at baseline and at end point. The proportion of health facilities in the study LGA with correctly filled HMIS 001 forms increased from 2 (18.2%) at baseline to 6 (54.5%) at post-intervention. Only one health facility (9.1%) had correctly filled HMIS form 001 at both baseline and end point in the control LGA (Table 1).

**Table 1:** Comparison of baseline and end point completeness and correctness of data in health facilities in study and control LGAs

Variables	Study LGA		Control LGA	
	Baseline Frequency (%)	End point Frequency (%)	Baseline Frequency (%)	End point Frequency (%)
**Data completeness**				
**OPD register**	1 (9.0)	4 (36.4)	0 (0.0)	0 (0.0)
Fisher’s exact	p=0.310			
**ANC register**	0 (0.0)	3 (27.3)	1 (9.1)	1 (9.1)
Fisher’s exact	p=0.214		p=1.000	
**Immunization register**	0 (0.0)	3 (27.3)	0 (0.0)	0 (0.0)
Fisher’s exact	p=0.214			
**HMIS form 001**	1 (9.1)	4 (36.4)	1 (9.1)	1 (9.1)
Fisher’s exact	p=0.310		p=1.000	
**Data correctness**				
**OPD register**	0 (0.0)	2 (18.2)	0 (0.0)	0 (0.0)
Fisher’s exact	p=0.476			
**ANC register**	2 (18.2)	5 (45.5)	0 (0.0)	0 (0.0)
Fisher’s exact	p=0.361			
**Immunization register**	0 (0.0)	3 (27.3)	0 (0.0)	0 (0.0)
Fisher’s exact	p=0.214			
**Form 001**	2 (18.2)	6 (54.5)	1 (9.1)	1 (9.1)
Fisher’s exact	p=0.182		p=1.000	

LGAs: local government areas, OPD: out patients department, ANC: antenatal clinic

At end point, there was an increase in the accuracy rate from baseline levels in the study health facilities. The increase was from 59.8% to 87.5% for the OPD register, 75.0% to 93.6% for the ANC register and from 68.0% to 88.8% in immunization register. The increase was statistically significant in all 3 registers. At end point, there was a decrease in the accuracy rates in all 3 registers in the control health facilities. The decrease was not statistically significant (Table 2).

**Table 2:** Comparison of baseline and end point accuracy rate of data in health facilities, in study and control LGAs

Variable	Study LGA		Control LGA	
	Baseline %	End point %	Baseline %	End point %
**Accuracy rate**				
**OPD register**	59.8	87.5	76.9	71.4
**Chi square**	15.400, p<0.001		0.520, p=0.471	
**ANC register**	75.0	93.6	77.6	71.4
**Chi square**	5.980, p=0.014		0.152, p=0.902	
**Immunization register**	68.2	86.0	73.3	72.7
**Chi square**	8.28, p=0.004		0.105, p=0746	
**Overall accuracy rate**	68.0	88.8	74.7	73.9
**Chi square**	29.100, p<0.001		0.316, p=0.859	

LGAs: local government areas, OPD: out patients department, ANC: antenatal clinic

At baseline, none of the health facilities in the study LGA analyzed their data. This increased to 4 (36.4%) at end point. The increase was not statistically significant. Presentation of data was done by 75% of the health facilities that analyzed them. There was no health facility in the control LGA that analyzed or presented their data either at baseline or at end point (Table 3). At end point, completeness of reporting in the study health facilities increased to 90.9% from the baseline level of 54.5%. The increase was statistically significant (p=0.02). That of the control health facilities increased to 48.5% from the baseline level of 45.5%. The increase was not statistically significant (p=0.453) (Table 3).

**Table 3:** Comparison of baseline and end point analysis, presentation, reporting of data and feedback in health facilities in study and control LGAs

Variables	Study LGA		Control LGA	
	Baseline Frequency (%)	End point Frequency (%)	Baseline Frequency (%)	End point Frequency (%)
**Analyze data (n=11)**	0 (0.0)	4 (36.4)	0 (0.0)	0 (0.0)
**Fisher’s exact**	p=0.090			
**Completeness of reporting (n=33)**				
**Reports received**	18	30	15	16
**Percentage completeness**	54.5%	90.9%	45.5%	48.5%
**Chi square**	5.000, p=0.025		0.117, p=0.731	
**Timeliness of reporting (n=33)**				
**Timely Reports**	15 (45.5)	24 (72.7)	15 (45.5)	12 (36.4)
**Chi square**	16.333, p<0.001		0.5641, p=0.4526	
**Feedback (n=11)**				
**Effort made**	0 (0.0)	6 (54.5)	0 (0.0)	0(0.0)
**Fisher’s exact**	p=0.012			

LGAs: local government areas

At end point, timeliness rate of reporting in the study health facilities increased to 72.7% from the baseline level of 45.5%. That of the control health facilities decreased to 36.4% from the baseline level of 45.5%. The changes in both the study and control health facilities were not statistically significant (Table 3). At baseline there was no effort made by the health facilities in both study and control LGAs to receive feedback on the data sent to the LGA health department.

At end point, 6 (54.5%) of the health facilities in the study LGA requested for feedback via visits and this was statistically significant (p=0.012). The health facilities in the control LGA did not make any effort to get feedback. At baseline, feedback was not received by any health facility in the study LGA but at end point all the health facilities had received feedback on data sent. The health facilities in the control LGA did not receive feedback on data sent either at baseline or at end point (Table 3).

At baseline, no health facility in the study LGA used data. At post-intervention, of the 4 (36.4) health facilities that analyzed their data, 2 (50.0%) used it for drug procurement, 3 (75.0%) for monitoring patient attendance and all 4 (100.0%) for health education and ordering vaccines. The increase was not statistically significant.

## Discussion

In this study the mean age of the healthcare workers was 34.6 ± 8.3 years for the study group and 33.7 ± 8.4 years for the control group and this is largely expected because they are in the working population group. During the baseline FGD, participants said they encountered difficulty in filling HMIS registers. Some of them said they didn't understand some variables in the registers and so leave the space provided for collecting them blank while others fill what they assume is being asked for. A participant said *"what I think is being asked for might not be what my colleague thinks is being asked for, so everybody just fills in what they assume is required"*. One participant also said *"some of the columns are too small for the information they are meant to contain and so in squeezing it into the space provided the writing may sometimes be illegible and transferring the data into summary forms becomes really difficult"*. After the intervention there was an increase in the percentage of health centers in the study LGA with completely and correctly filled HMIS registers and health facility summary forms from the baseline levels. The increase was not as high as the one observed in a study done in Enugu, Nigeria on enhancing the data management skills of PHC workers [17]. This could be because majority of the health workers in this study have never been trained on HMIS while almost half of the respondents in the Enugu study had been trained previously and this was a retraining. There was no health facility with completely or correctly filled registers both at baseline and end point in the control LGA. Only 9.1% had completely and correctly filled health facility summary form. The improvement in the study LGA could be attributed to the training. This can be corroborated by the FGD findings. At baseline in both LGAs they said tools were given to them without training them on how to use them. A participant said *"only a few staff know how to manage data but most of us do not know how to because we have not been trained. Very few have the opportunity of being trained"*. At end point in the study LGA they noted that their data management skills had improved. A participant said *"our skills are better now because we have been trained. Our skills show in the way we fill the registers. The feedback we got from the local government monitoring and evaluation officer was that the quality of the data reported had improved"*. Another participant said *"now I can do a little analysis that helps us to monitor patients' attendance and choose topics for health education in my health centre"*.

In the study LGA, the completeness of facility reporting increased from 54.5% at baseline to 90.9% at end point. The increase was statistically significant. The completeness rate in this study was not up to 100% as was seen in a study in Rwanda [25]. This could be attributed to the interventions implemented in the country by the Rwandan government and NGOs to strengthen the health system and improve data quality, which were performance-based, financing, change in technology from locally based system to a web based system, training on how to use the system and data cleaning done at the health facility level [25]. That of the control LGA in this study also increased marginally (from 45.5% to 48.5%). This increase might be due to the baseline data collection process which might have gingered them and led to this marginal increase. Poor reporting will discourage use of data generated for decision making and policy formulations. There was an increase in the timeliness of reporting of the study health facilities in this study but the increase was not statistically significant, which contrasts with the finding in a study done in Uganda on strengthening district based health reporting through health management information software system (DHIS) [26]. This could be because the health facilities in this study use paper-based reporting system and had to physically take the report to the LGA health department which involves transportation which may not be always available. This same reason could also account for the contrast in the timeliness of reporting observed between this study and a study in Mayuge, Uganda [27] where record assistants were facilitated to go to the health facilities to collect the reports as opposed to the health workers taking them to the health department of the LGA as was the case in this study. At the baseline FGD, the participants did not think it mattered if the data reporting is timely or not but at end point they said they now make more effort to report on time because they now know the importance of timely reporting.

There was no health facility in the study LGA that analyzed data at baseline. This increased to 4 (36.4%) after the intervention, of which 3 (75%), presented their information. Even though the increase in facilities that analyzed and presented their data in the study group were not statistically significant it showed an improvement compared to the control group where no health facility was found to have analyzed their data either at the beginning or at the end of the study. The baseline finding could be attributed to the respondents' lack of skills in data analysis. In the words of a participant during an FGD session *“most of us don't know how to analyze data”*. Data use is likely to improve if staff have the skills in analysis and interpretation of data and also have an understanding of how and why data should be used. At baseline, none of the health facilities in the study or control areas analyzed their data. After the intervention, 4 (36.4%) of the study health facilities analyzed their data. All four facilities used it for health education and ordering vaccine, two for drug procurement (50.0%) and three (75%), for monitoring patient attendance. At FGD at baseline, most participants said they didn't use data locally because they didn't know they were supposed to use them. As one participant said *“we are just to collect the data and send it to the M and E officer in the LGA, they are the ones that use the data.* Another one said *“we keep a copy in the health facility to show guests like donor partners on request”*. In the words of one of the participants at the end point FGD in the study LGA “now that we know how to use the data that we generate, we use them to know the amount of vaccine to collect, the diseases that are common in the community and to know the number of under-fives that have completed their immunization.”

There was no feedback on the data forwarded to the LGA health authority in both the study and control group and no effort was made to get feedback at baseline. This remained the same in the control group at end point but all the health facilities in the study group received feedback. This could be because more than half (54.5%) of them requested for it from the health department of the LGA. This is in contrast with a study in Enugu, Nigeria, where feedback on the data forwarded to higher authority was received by 33.3% of the study and 22.2% of the control centers prior to the intervention. End point results showed that feedback improved to 55.6% in the study health centers [16]. Some of the health workers during the FGDs at baseline said apart from lack of training, the poor quality data generated could also be attributed to lack of feedback on the data reported. Some health workers at end point FGD attributed the feedback received by all the health facilities to the effort made by some of them. Feedback is an essential component of any reporting system, it recognizes the source from which the data is been generated and serves as a source of encouragement. Poor data management practice in health workers could lead to poor quality data which will invariably lead to inappropriate decision making in healthcare system.

**Limitation of the study:** The timeliness rate of reporting of the health facilities was meant to be ascertained from the health department of the LGA but this could not be done due to the absence of records on when health facilities submitted their monthly summaries. The timeliness rate was calculated instead using the time of data remission recorded in the reports of the officers-in-charge of the PHCs.

## Conclusion

In the study LGA, accuracy rate, completeness of reporting, timeliness rate of reporting and feedback increased after the intervention by 20.8%, 36.4%, 27.2% and 54.5% respectively. The increase were all statistically significant. There was improvement in data completeness, analysis, presentation and local use of information though not statistically significant. In the control group there was generally no improvement in data management practice. Thus the training intervention was effective in improving the data management practice on HMIS in the study group. Therefore, in order to improve the data management practice of health workers in-service training and re-training should be done.

### What is known about this topic

- One of the problems encountered in the implementation of health management information system is the lack of skilled personnel and severely inadequate trained workforce especially at the primary health care facility level;
- There is incomplete, untimely and largely incorrect reporting of data and grossly inadequate capacity to analyze and utilize data.

### What this study adds

- The baseline findings in this study corroborate findings in previous studies which showed poor data management practices in health management information system among health care workers. There was incomplete, incorrect and untimely reporting of data and also an inability to analyze and use data;
- Complete, accurate and timely data and the capacity to use information lead to decisions that improve health by improving the health system's ability to respond to health needs at all levels. Strengthening data management at primary health care level amounts to strengthening the health care delivery system in the country;
- This study showed that a training intervention can help improve data management practice in health management information system; training and re-training could be difficult to sustain especially in a developing country like Nigeria with limited resources.

## Competing interests

The authors declare no competing interests.
